# Single coronary artery with aortic valve replacement followed by aortic root replacement due to endocarditis: A case report

**DOI:** 10.1002/ccr3.8817

**Published:** 2024-05-03

**Authors:** Makoto Taoka, Akira Sezai, Masanao Ohba, Yoshiki Kitazumi, Taisuke Hanamura, Yasuo Okumura, Masashi Tanaka

**Affiliations:** ^1^ Department of Cardiovascular Surgery Nihon University School of Medicine Tokyo Japan; ^2^ Division of Cardiology, Department of Medicine Nihon University School of Medicine Tokyo Japan

**Keywords:** freestyle valve, prosthetic valve endocarditis, single coronary artery

## Abstract

A woman with a single coronary artery underwent aortic valve replacement due to aortic stenosis. Two years later, she developed an aortic annular abscess around the right coronary cusp and non‐coronary cusp. Significant adhesions to the right coronary artery (RCA) resulted from the abscess, making artery separation challenging, and raising concerns about potential future RCA stenosis. The patient subsequently underwent aortic root replacement and coronary artery bypass grafting. Utilizing a freestyle valve and a saphenous vein graft for the RCA. Following the procedure, the patient was discharged and has remained symptom‐free without any recurrence of infection for 2 years.

## Graphical abstract

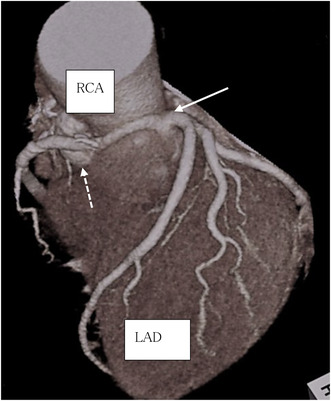

## 1 INTRODUCTION

Single coronary artery is relatively rare among congenital coronary anomalies, with an incidence rate reported to be approximately 0.04% to 0.06% based on coronary angiography results.^1^, ^2^ This study presents a case in which an abscess formed in the aortic valve annulus 2 years after aortic valve replacement (AVR) in a patient with single coronary artery (Lipton L II‐B). The patient underwent aortic root replacement using a Freestyle valve and coronary artery bypass surgery.

In this case, due to severe adhesions, separation of the right coronary artery (RCA) was challenging during the creation of the Carrel patch, the risk of future inflammatory spread, and coronary artery stenosis. Therefore, a bypass was performed using the great saphenous vein. Additionally, perfusion abnormalities in the left coronary artery (LCA) were observed, likely due to the severe adhesions at the aortic root and bending of the Carrel patch, necessitating an additional coronary artery bypass.

Coronary revascularization in single coronary artery, especially in cases with severe adhesions, requiring careful consideration of treatment strategies. This report aims to contribute to the decision‐making process for future treatment strategies by providing an approach to treating such complex cases.

## 2 CASE HISTORY

A 76‐year‐old woman underwent AVR with a bioprosthetic valve (CEP Magna 21 mm; Edwards Life Sciences, Irvine, CA, USA) due to aortic valve stenosis. Postoperatively, the patient developed complete atrioventricular block and required pacemaker implantation. During her outpatient follow‐up after discharge, she did not experience heart failure or infections. However, she visited her outpatient clinic 2 years later with fever and general fatigue. Significant elevations in inflammatory markers were observed (WBC18.3 × 10^3^/μL and CRP 18.6 mg/dL, respectively), and *Staphylococcus capitis* was detected in her blood cultures. Additionally, CT tomography revealed renal and splenic infarctions, and an aortic valve abscess was suspected on transesophageal echocardiography. Infective endocarditis was diagnosed, and a 6‐week course of vancomycin successfully resolved the infection. The patient was transferred to our hospital for surgical removal of the aortic annulus abscess.

### 2.1 Diagnostic assessments

**FIGURE 1:**
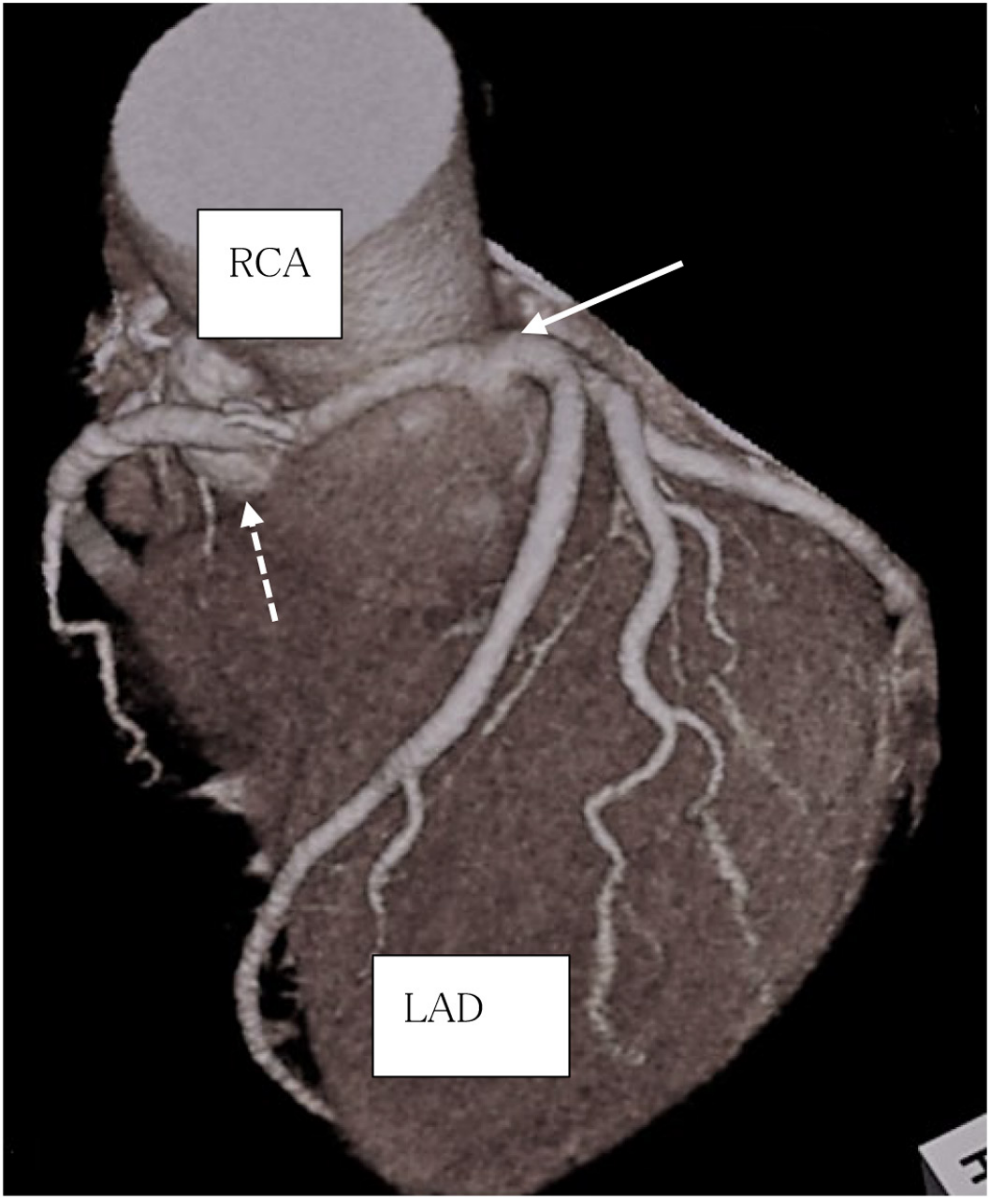
Preoperative CT. (1) right coronary artery originates from the left coronary artery(←). (2) abscess cavity is in contact with the RCA (#1). LAD, left anterior descending arteryl; RCA, right coronary artery.

Upon admission, her height, body weight, and body temperature were 149 cm and 53 kg, and 36.4°C, respectively. Her blood pressure was 92/43 mmHg, with a regular pulse of 60 beats/min. Inflammatory markers had normalized (WBC 7.90 × 10^3^/μL and CRP 0.23 mg/dL, respectively), and procalcitonin level were negative. The left ventricular ejection fraction was 81%. However, aortic valve insufficiency originating from the same site as the suspected abscess cavity around the right coronary cusp‐non‐coronary cusp (paravalvular leakage) was observed without apparent vegetation. No significant stenosis was observed in the coronary artery, and the RCA shared a common opening with the LCA originating from the left coronary cusp (LCC). The RCA ran anteriorly to the aorta between the aorta and pulmonary artery, with an abscess observed adjacent to the dorsal side of the RCA (#1) (Figure 1A, B).

### 2.2 Treatment

**FIGURE 2:**
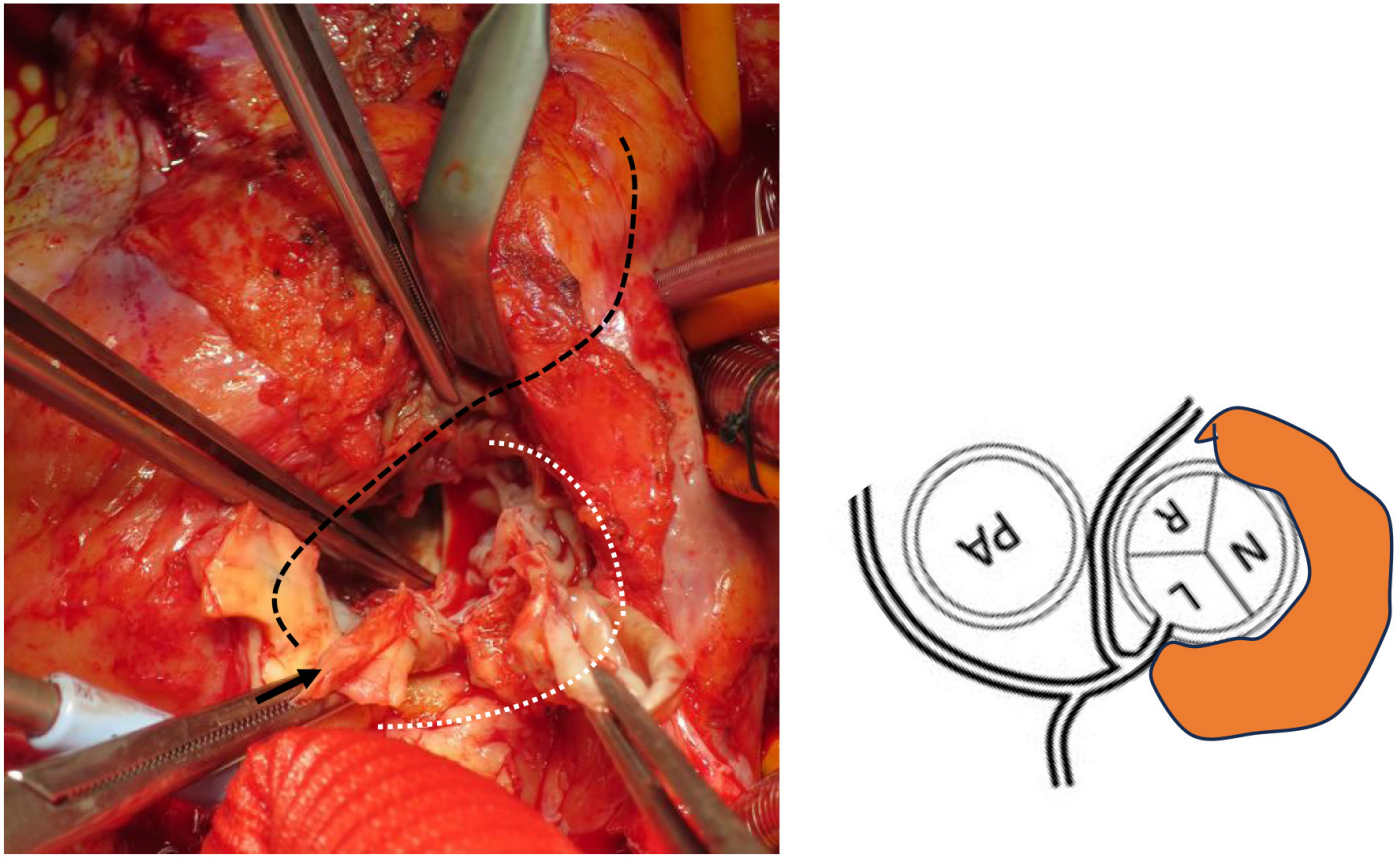
After prosthetic valve resection. The abscess cavity is centered on the non‐coronary cusp, just below the entrance of the left coronary cusp, and in the direction of the right coronary cusp where it is released to 12 o'clock (white dotted line and orange field). The ostium of the coronary artery (→) and the right coronary artery running area (black dotted line) are also highly adherent. L, left coronary cusp; N, non coronary cusp; PA, pulmonary artery; R, right coronary cusp.

The right atrium, ascending aorta, and the anterior portion of the right ventricle exhibited significant adhesion and exfoliation. We established cardiopulmonary bypass by cannulating the superior and inferior vena cava and the ascending aorta. Cardioplegia was selectively injected into the LCA, inducing cardiac arrest with ease. Subsequently, retrograde coronary perfusion was performed every 20 min. Following coronary peripheral anastomosis under circulatory arrest, upon inspecting the aortic root, we noted partial dislocation of the previously replaced prosthetic valve at the non‐coronary cusp (NCC) annulus. During removal of the bioprosthetic valve, we identified the pannus tissue underneath the valve, and made extensive efforts to remove as much of it as possible. The abscess cavity was open, extending from the LCC just below the LCA ostium to the RCC at the 12 o'clock position, with the NCC at the center (Figure 2). While creating a Carrel patch for the LCA, we found that the RCA was running within the aortic wall, and was in close proximity to the abscess cavity located at the dorsal RCC. To prevent potential damage caused by detachment, we transected the RCA, and created a bypass using the great saphenous vein on RCA #2 to facilitate the management of the LCA orifice. The LCC and RCC still had a remaining annulus, and we used an everted mattress technique to secure the sutures to the annulus. However, as no annulus remained in the NCC, we placed a suture outside the aortic wall to secure it. We selected a 21 mm Freestyle valve and reconstructed the LCA using the Carrel patch method. After releasing the aortic blockage, we observed recurrent episodes of ventricular fibrillation, indicating a potential perfusion abnormality in the LCA. We performed coronary artery bypass grafting in the left anterior descending artery in the great saphenous vein. With the assistance of an intra‐aortic balloon pump (IABP), we safely discontinued cardiopulmonary bypass, and the patient was successfully transferred back to the intensive care unit. The total operating time was 565 min, with a cardiopulmonary bypass time of 366 min and an aortic clamping time of 231 min.

### 2.3 Follow‐up

Given the stable hemodynamic status of the patient, we removed the IABP on the same day as the surgery. Postoperatively, administration of daptomycin (300 mg/day) for 4 weeks improve the inflammatory response. No bacteria were detected in abscess tissues obtained during surgery. Postoperative CT tomography confirmed graft patency. On the 32nd day after the surgery, the patient was transferred to the hospital without any abnormalities in the Freestyle valve. No sign of infection recurrence has been observed for 2 years.

## 3 DISCUSSION

Single coronary artery disease is a rare congenital coronary artery malformation, with a reported incidence of 0.04% to 0.06% based on coronary angiography.^1^, ^2^ The Lipton classification is commonly used to classify this disease.^3^ In our case, the RCA formed a common opening in the LCC and coursed within the anterior aspect of the aortic wall before following a normal trajectory, corresponding to classification II‐B in the Lipton classification system. Patients with a single coronary artery disease often have a high incidence of congenital heart disease, including aortic bicuspid valve, tetralogy of Fallot, and macrovascular dislocation, seen in 40% of cases.^4^ However, in this case of single AVR, at the age of 76 years, the findings revealed a tricuspid aortic valve and no other anatomical abnormalities, except for the presence of a common orifice where the RCA originated from the LCC. Notably, even in cases without cardiac malformations, patients with single coronary artery disease have reported ischemic heart disease, sudden death, heart failure, and conduction disorders.

The pathogenesis of ischemia in such cases may be attributed to factors such as compression due to the artery running between the aorta and pulmonary artery, or the origin of the coronary artery being at an acute angle, leading to conditions such as slit‐like entrance stenosis caused by the artery running within the aortic wall.^5^

In this case, involving a single coronary artery disease classified as Lipton L II‐B, where the coronary artery ran with a risk of ischemia. There was no evidence of myocardial ischemia during the initial AVR surgery or preoperative assessment for the current surgery. Because of the inability to evaluate patient condition at that time, bypass surgery was not initially considered. However, during creation of the Carrel patch, we encountered significant adhesions caused by an abscess at the annulus, it difficult to separate the RCA. Additionally, the distal portion of the RCA #1 adhere to the abscess cavity, raising concerns regarding potential coronary artery stenosis due to inflammation. Therefore, we bypassed the RCA using the great saphenous vein. Furthermore, because of suspected abnormal perfusion of the LCA reconstruction, indicated by ventricular fibrillation during cardiopulmonary bypass, we performed bypass using the great saphenous vein to the left anterior descending artery. This might have been attributed to flexion of the Carrel patch caused by adhesion of the aortic root. In cases where severe adhesions are suspected during reoperation for single coronary artery disease, considering Piehler reconstruction might be more beneficial to prevent such adverse events. In this rare case, the choice of the bypass graft was crucial. The RCA has the potential to cause ischemia due to compression between the aorta and pulmonary artery. Unlike stenosis caused by arteriosclerosis, this type of narrowing usually allows the maintenance of blood flow in the coronary artery. When considering the internal thoracic artery as a graft option, there is a risk of flow competition and the need for additional revascularization.^6^ Therefore, we opted to use the great saphenous vein as the bypass graft. However, even in bypass procedures using the great saphenous vein, several cases of occlusion have been reported, with reports highlighting the advantages of the unroofing method.^7^ In this case, we performed CT and myocardial scintigraphy 2 years after surgery to confirm graft patency and rule out ischemia. Nevertheless, strict follow‐ups are necessary in the future.

## 4 CONCLUSION

An aortic annular abscess developed following AVR in a patient with single coronary artery. This was successfully treated with aortic root replacement using the Freestyle valve and coronary artery bypass grafting. Revascularization of a single coronary artery, especially when severe adhesions are present, is a challenging procedure that necessitates the choice of an appropriate treatment strategy.

## AUTHOR CONTRIBUTIONS

**Makoto Taoka:** Conceptualization. **Akira Sezai:** Conceptualization. **Masanao Ohba:** Conceptualization. **Yoshiki Kitazumi:** Conceptualization. **Taisuke Hanamura:** Conceptualization. **Yasuo Okumura:** Conceptualization. **Masashi Tanaka:** Conceptualization.

## FUNDING INFORMATION

No funding sources.

## CONFLICT OF INTEREST STATEMENT

The authors declare that they have no conflict of interest.

## ETHICS STATEMENT

This case report has observed the ethical guidelines.

## CONSENT

Written informed consent was obtained from the patient for publication of this case report and accompanying images. A copy of the written consent is available for review by the Editor‐in‐Chief of this journal on request.

## Data Availability

The data that support the findings of this study are available from the corresponding author upon reasonable request.
